# The impact of dialysate flow rate on haemodialysis adequacy: a systematic review and meta-analysis

**DOI:** 10.1093/ckj/sfae163

**Published:** 2024-06-04

**Authors:** Yasmin Iman, Ryan Bamforth, Ruth Ewhrudjakpor, Paul Komenda, Kelley Gorbe, Reid Whitlock, Clara Bohm, Navdeep Tangri, David Collister

**Affiliations:** Seven Oaks Hospital Chronic Disease Innovation Centre, Winnipeg, Manitoba, Canada; Seven Oaks Hospital Chronic Disease Innovation Centre, Winnipeg, Manitoba, Canada; Seven Oaks Hospital Chronic Disease Innovation Centre, Winnipeg, Manitoba, Canada; Seven Oaks Hospital Chronic Disease Innovation Centre, Winnipeg, Manitoba, Canada; Quanta Dialysis Technologies, Alcester, UK; University of Manitoba, Rady Faculty of Health Sciences, Department of Internal Medicine, Winnipeg, Manitoba, Canada; Quanta Dialysis Technologies, Alcester, UK; Seven Oaks Hospital Chronic Disease Innovation Centre, Winnipeg, Manitoba, Canada; Seven Oaks Hospital Chronic Disease Innovation Centre, Winnipeg, Manitoba, Canada; University of Manitoba, Rady Faculty of Health Sciences, Department of Internal Medicine, Winnipeg, Manitoba, Canada; Seven Oaks Hospital Chronic Disease Innovation Centre, Winnipeg, Manitoba, Canada; University of Manitoba, Rady Faculty of Health Sciences, Department of Internal Medicine, Winnipeg, Manitoba, Canada; Seven Oaks Hospital Chronic Disease Innovation Centre, Winnipeg, Manitoba, Canada; University of Manitoba, Rady Faculty of Health Sciences, Department of Internal Medicine, Winnipeg, Manitoba, Canada; University of Alberta, Faculty of Medicine & Dentistry, Department of Medicine, Edmonton, Alberta, Canada

**Keywords:** adequacy, dialysate flow rate, haemodialysis, Kt/V, urea reduction ratio

## Abstract

**Background:**

Patients with kidney failure treated with maintenance haemodialysis (HD) require appropriate small molecule clearance. Historically, a component of measuring ‘dialysis adequacy’ has been quantified using urea kinetic modelling that is dependent on the HD prescription. However, the impact of dialysate flow rate on urea clearance remains poorly described *in vivo* and its influence on other patient-important outcomes of adequacy is uncertain.

**Methods:**

We searched Embase, MEDLINE and the Cochrane Library from inception until April 2022 for randomized controlled trials and observational trials comparing a higher dialysate flow rate (800 ml/min) and lower dialysate flow rate (300 ml/min) with a standard dialysis flow rate (500 ml/min) in adults (age ≥18 years) treated with maintenance HD (>90 consecutive days). We conducted a random effects meta-analysis to estimate the pooled mean difference in dialysis adequacy as measured by Kt/V or urea reduction ratio (URR).

**Results:**

A total of 3118 studies were identified. Of those, nine met eligibility criteria and four were included in the meta-analysis. A higher dialysate flow rate (800 ml/min) increased single-pool Kt/V by 0.08 [95% confidence interval (CI) 0.05–0.10, *P* < .00001] and URR by 3.38 (95% CI 1.97–4.78, *P* < .00001) compared with a dialysate flow rate of 500 ml/min. Clinically relevant outcomes including symptoms, cognition, physical function and mortality were lacking and studies were generally at a moderate risk of bias due to issues with randomization sequence generation, allocation concealment and blinding.

**Conclusion:**

A higher dialysate flow increased urea-based markers of dialysis adequacy. Additional high-quality research is needed to determine the clinical, economic and environmental impacts of higher dialysate flow rates.

KEY LEARNING POINTS **What was known:**

- Achieving minimum Kt/V targets has been shown to improve patient morbidity and mortality.
- There are many other clinically relevant outcomes related to dialysis adequacy, including blood pressure, volume status, ultrafiltration rate, inflammation, middle molecule clearance, physical function, cognition and a variety of patient-reported outcome measures.
- Understanding how to optimize Kt/V by modifying the HD prescription is important, but not all approaches may be feasible when considering patient values and preferences.

**This study adds:**

- An understanding of the impact of higher and lower dialysate flow rates, compared with the standard dialysis flow rate of 500 ml/min, on dialysis adequacy as measured by Kt/V, urea reduction ratio and patient-important outcomes, including symptoms, cognition, physical function and quality of life, is needed.

**Potential impact:**

- Increasing dialysis flow rates in adults receiving maintenance HD improves dialysis adequacy as measured by urea kinetic modelling metrics, including URR and single-pool Kt/V.
- More evaluation is necessary in high-quality prospective studies.

## INTRODUCTION

Patients with kidney failure undergoing maintenance haemodialysis (HD) require adequate removal of solutes and fluid in order to restore homeostasis. Historically, one key marker of ‘dialysis adequacy’ has focused on urea kinetic modelling quantified by dialyzer clearance of urea (K) multiplied by the duration of the dialysis session (t) adjusted for the volume (V) of distribution [1]. Achieving minimum Kt/V targets have been shown to improve patient morbidity and mortality, but dialysis adequacy is a complex construct that encompasses many traditional targets, including small molecule clearance, potassium, acid–base status, anaemia, mineral and bone disorder and cardiovascular risk [2–6]. In addition, there are many other clinically relevant outcomes related to dialysis adequacy, including blood pressure (BP), volume status, ultrafiltration rate, inflammation, middle molecule clearance, physical function, cognition and a variety of patient-reported outcome measures (PROMs) related to symptoms that are not captured by Kt/V [7]. However, Kt/V is still an important metric in delivering safe and quality HD care and remains an indicator of quality of care for dialysis programs.

Understanding how to optimize Kt/V by modifying the HD prescription is important [2–7], but not all approaches may be feasible when considering patient values and preferences, reimbursement constraints and scheduling of facility-based HD treatments. Contemporary HD systems offering more portability and improved usability for self-care are becoming increasingly available [8–13]. These systems unlock the potential for HD to be performed in a range of settings and a diverse group of users. Some HD systems, due to either the use of batch-based or bagged dialysate or limitations of water systems, are unable to attain dialysate flow rates at or above the standard rate of 500 ml/min provided by conventional HD systems [8, 9]. Whether this limitation affects the ability of the patient to achieve an optimal Kt/V target is unclear and the relative impact of higher or lower dialysate flow rates on dialysis adequacy in clinical settings is unknown.

We performed a systematic review and meta-analysis to summarize the impact of higher and lower dialysate flow rates,

compared with the standard dialysis flow rate of 500 ml/min, on dialysis adequacy as measured by Kt/V, urea reduction ratio (URR) and patient-important outcomes, including symptoms, cognition, physical function and quality of life.

## MATERIALS AND METHODS

### Data sources and searches

We developed and followed a protocol that considered population, intervention, comparison and outcome criteria for our search strategy. The population of interest was adults (age ≥18 years) with kidney failure on maintenance HD (dialyzing for ≥90 consecutive days). The setting of HD could have been either facility-based or home HD. Studies of interest contained interventions that were a low dialysate flow rate (300 ml/min) or a high dialysate flow rate (800 ml/min) and a comparator of the standard dialysate flow rate (500 ml/min). Any type of HD prescription was permitted, including weekly schedule, duration, vascular access, blood flow rate and dialyzer. Studies considering dialysate flow rates not equal to 300, 500 or 800 ml/min were included but not meta-analysed. Our primary outcome was adequacy of dialysis measured by URR or Kt/V quantified using either single-pool (spKt/V), double-pool (dpKt/V) equilibrated (eKt/V) or standard (stdKt/V) [14–19]. Our secondary outcomes included all-cause mortality and the following PROMs: health-related quality of life as measured by the EuroQol-5 Dimension (EQ-5D), Kidney Disease Quality of Life (KDQoL-36) questionnaire and its short form version (KDQoL-SF); symptom burden as measured by the Edmonton Symptom Assessment Scale (ESAS) or its renal version (ESAS-r); and other disease-specific instruments including but not limited to mood, pruritus, restless legs, gastrointestinal symptoms (nausea, vomiting, appetite), fatigue, cognition (e.g. Mini Mental Status Exam, Montreal Cognitive Assessment) and physical function (e.g. 6-minute walk test, sit to stand, hand grip strength, others). We applied methodological approaches outlined in the Preferred Reporting Items for Systematic Reviews and Meta-Analyses (PRISMA) guidelines as well as those outlined in PRISMA-S (literature search extension) [20, 21]. This study was registered on PROSPERO (CRD42022321877) [22].

**Figure 1:**
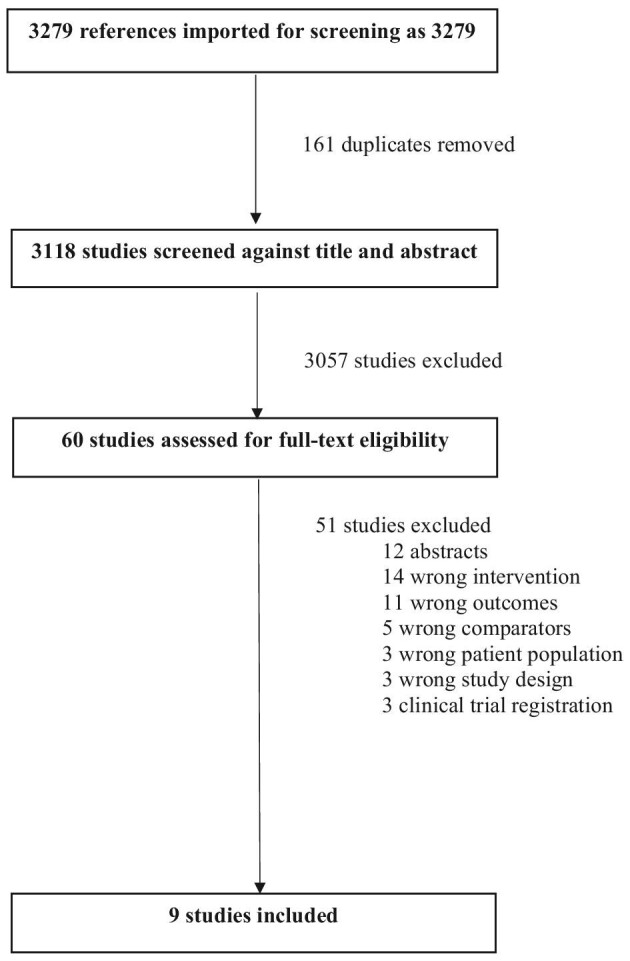
PRISMA flow diagram.

In collaboration with a medical librarian, original research articles were identified from the following databases: Embase, MEDLINE and the Cochrane Library. We included articles in the English language only, there was no restriction on sample size and grey literature was excluded (Fig. 1). Our search strategy included keywords and MeSH terms related to ‘dialysis adequacy’, ‘haemodialysis adequacy’, ‘clearance’, ‘urea reduction ratio’, ‘Kt/V’, ‘dialysis dose’ and ‘dialysate flow’. We included randomized controlled trials (RCTs) and observational studies; all other study designs were excluded.

Two reviewers (Y.I. and R.E.) independently reviewed each article by title and abstract to select articles for full-text review. The two reviewers (Y.I. and R.E.) then screened the full text of the selected articles to determine if they were eligible for inclusion in the analysis. References of the included studies were also screened for additional studies that met the inclusion criteria. All disagreements were resolved by consensus after consultation with a third reviewer (D.C.).

### Data extraction and quality assessment

A data extraction form was created to capture relevant information from the studies that met the inclusion criteria. Two reviewers (Y.I. and R.E.) independently extracted the data from the studies. The following data were extracted from the included studies, depending on availability: article information (title, first author, year, journal, title, location, design), inclusion and exclusion criteria, characteristics of the intervention and comparator groups (sample size, sex/gender, age, vascular access type, HD setting, study duration, primary and secondary outcome measures. Outcomes from studies with multiple arms related to differing baseline HD prescriptions (i.e. weekly schedule, duration, vascular access, blood flow rate and dialyzer) were extracted separately if present and both unscaled and scaled Kt/V outcomes were reported if applicable [23].

Additionally, two reviewers (Y.I. and R.E.) assessed the quality of the included studies using the Cochrane Risk of Bias Tool [24]. This tool consists of six domains (sequence generation, allocation concealment, blinding, incomplete outcome data, selective outcome reporting and ‘other’ sources of bias) and a categorization of the overall risk of bias. Each separate domain was rated as low, unclear or high risk. The non-randomized trials were assessed using the ROBINS-I (Risk Of Bias In Non-randomized Studies – of Interventions) tool. Discrepancies between the two reviewers were resolved with a third reviewer (D.C.) by consensus.

### Data synthesis and analysis

We performed random effects meta-analysis employing the generic inverse variance method to estimate mean differences and related 95% confidence intervals (CIs). Statistical heterogeneity was quantified using the *I*^2^ statistic, with statistical significance assessed with the χ^2^ test. Methods outlined in the Cochrane Handbook for Systematic Reviews of Interventions were used to calculate standard deviations (SDs) of the difference, standard errors of the mean (SEM) difference and the mean difference when required [25]. We assumed a correlation coefficient value of 0.5 in the main analyses, with sensitivity analyses considering values of 0.25 and 0.9. In instances where outcome-specific SDs were not available, we imputed the SEM difference based on values from the other included studies. This was only performed when the minority of studies in the meta-analysis required imputation. As exploratory analyses, we performed a random effects network meta-analysis estimating mean differences and 95% CIs for the outcome spKt/V. The standard dialysate flow rate (500 ml/min) was compared with those both greater and less than 500 ml/min. For main and exploratory analyses, two or more studies with the relevant outcome were required. Statistical significance was determined using α = 0.05.

Statistical analysis pertaining to the standard meta-analysis was performed using Review Manager (RevMan version 5.3, Nordic Cochrane Center, Copenhagen, Denmark). Network meta-analysis was performed using the netmeta package in R version 4.1.0 (R Foundation for Statistical Computing, Vienna, Austria) [26].

## RESULTS

### Study selection

The PRISMA flow diagram is presented in Fig. 1. The initial search strategy yielded 3118 research articles eligible for screening (3279 total minus 161 duplicates). Of those, 361 were selected for full-text review, with 9 meeting the inclusion criteria [7, 27–34]. Of the nine studies, three were randomized crossover trials and six were observational studies (prospective crossover trials). The RCTs were set in Canada (*n* = 1), Colombia (*n* = 1) and the USA (*n* = 1) and the remaining six observational studies were set in Germany (*n* = 1), Greece (*n* = 1), Egypt (*n* = 2), Morocco (*n* = 1) and Spain (*n* = 1). Only two of the nine (22%) included studies were >3 months in duration. Primary outcomes that were reported were Kt/V (unspecified type), spKt/V, Kt, eKt/V and URR. Secondary outcomes that were reported were body weight, BP, nutritional status, anaemia and quality of life. All studies included patients with either an arteriovenous fistula or a central venous catheter. The type of vascular access and blood flow rate were kept constant during the crossover period. Study characteristics are described in Tables 1 and 2.

**Table 1:** Study characteristics.

				Dialysis dose					Outcome	
Author (year)	Country	Study design	Age (years)	Intervention	Comparator	*n* (control/intervention)	HD setting	Duration of trial	Primary	Secondary
Ahrenholz *et al.* (2015) [27]	Germany	Observational crossover trial	Adult population	a. 300 ml/minb. 800 ml/min	500 ml/min	18 (18/18)	HD	3 weeks	spKt/V	NR
Alayoud *et al.* (2012) [28]	Morocco	Single-centre observational crossover trial	49 ± 17	700 ml/min	500 ml/min	33 (33/33)	HD	3 weeks	Kt/V^a^]	NR
Albalate *et al.* (2015) [29]	Spain	Single-centre observational crossover trial	78	a. 400 ml/minb. 700 ml/min	500 ml/min	31 (31/31)	HD	27 weeks	Kt (L)	NR
Azar, Ahmad (2009) [7]	Egypt	Observational crossover trial	50.51 ± 15.12	800 ml/min	500 ml/min	138 (138/138)	HD	32.14 ± 28.72 months	spKt/V, URR	NR
Azar *et al.* (2007) [30]	Egypt	Observational crossover trial	48.21 ± 13.38	800 ml/min	500 ml/min	134 (134/134)	HD	3 months	spKt/V, URR	Body weight
Molano-Trivino *et al*. (2019) [31]	Colombia	Crossover RCT	62.5	400 ml/min	500 ml/min	46	HD	5 years	Kt/V^a^	Body weight
Panagoutsos *et al*. (2001) [32]	Greece	Observational crossover trial	52.6 ± 14.5	560 ml/min	500 ml/min	34 (34/34)	HD	2 years	Kt/V^a^, URR	Body weight, BP, nutritional status, anaemia and QoL
Wang *et al*. (2008) [33]	Canada	Crossover RCT	56	800 ml/min	500 ml/min	18 (12/12)	HD	12 weeks	spKt/V	QoL
Ward *et al*. (2011) [34]	United States	Multicentre crossover RCT	50	800 ml/min	500 ml/min	33 (33/33)	HD	NR	spKt/V, eKt/V	NR

NR: not reported.

a Type of Kt/V was not specified.

**Table 2:** Study trial characteristics.

Author (year)	AVF/AVG	Dialysis schedule	Dialysis duration	QB	Dialyzer	Urea assays	Kt/V methodology
Ahrenholz *et al.* (2015) [27]	Catheter or AVF	2–3 times/week	3–4 hours	300 ml/min	FDY-150 GW, FX 60, VitaPES 150HF	NR	Q_B_, dialyzer and URR
Alayoud *et al.* (2012) [28]	Catheter or AVF	3 times/week	NR	300 ml/min	Fresenius 5008	NR	Online clearance monitoring
Albalate *et al.* (2015) [29]	Tunnelled central venous catheter or AVF	3 times/week	3–4 hours	374–382 ml/min	AK200, Fresenius 5008	NR	Recorded directly from monitor/system
Azar (2009) [7]	Catheter or AVF	3 times/week	3–4 hours	300 ml/min	Fresenius 4008B	NR	Daugirdas second-generation formula
Azar *et al.* (2007) [30]	Catheter or AVF	3 times/week	3–4 hours	200–350 ml/min	NR	NR	Daugirdas second-generation formula
Molano-Trivino *et al*. (2019) [31]	Catheter or AVF	3 times/week	4 hours	NR	NR	NR	NR
Panagoutsos *et al*. (2001) [32]	Catheter or AVF	3 times/week	4 hours	240–410 ml/min	NR	NR	Daugirdas second-generation formula
Wang *et al*. (2008) [33]	Catheter or AVF	3 times/week	4–4.5 hours	350–400 ml/min	F80A, Fresenius	Pre- and post-dialysis urea samples taken with arterial needle	Post-dialysis blood samples, pre-dialysis and post-dialysis urea samples through arterial needle Kt/V = {−ln(urea post/urea pre) + 3[(weight pre – weight post)/weight post]}/[1 − 0.01786(time dialysis)]
Ward *et al*. (2011) [34]	Catheter or AVF	3 times/week	3–4 hours	400–450 ml/min	Single-use Polyflux Revaclear, Revaclear MAX, Phoenix dialysis machines	Pre- and post-dialysis plasma urea BUN	spKt/V and eKt/V were calculated using pre- and post-dialysis BUN concentrations according to Daugirdas second-generation formula

AVF: arteriovenous fistula; AVG: arteriovenous graft; BUN: blood urea nitrogen; NR: not reported.

### Risk of bias assessment

**Figure 2:**
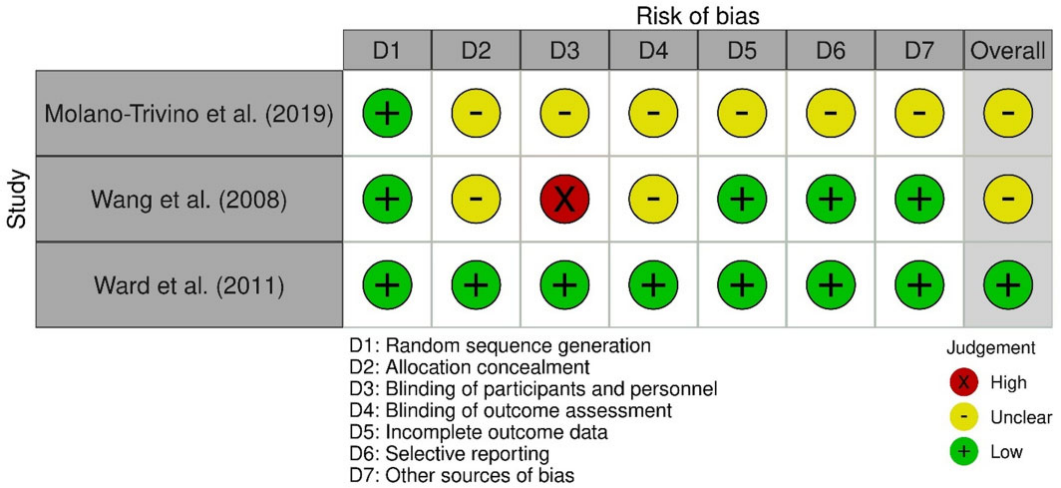
Risk of bias assessment for randomized trials using the revised Cochrane Risk of Bias Tool.

**Figure 3:**
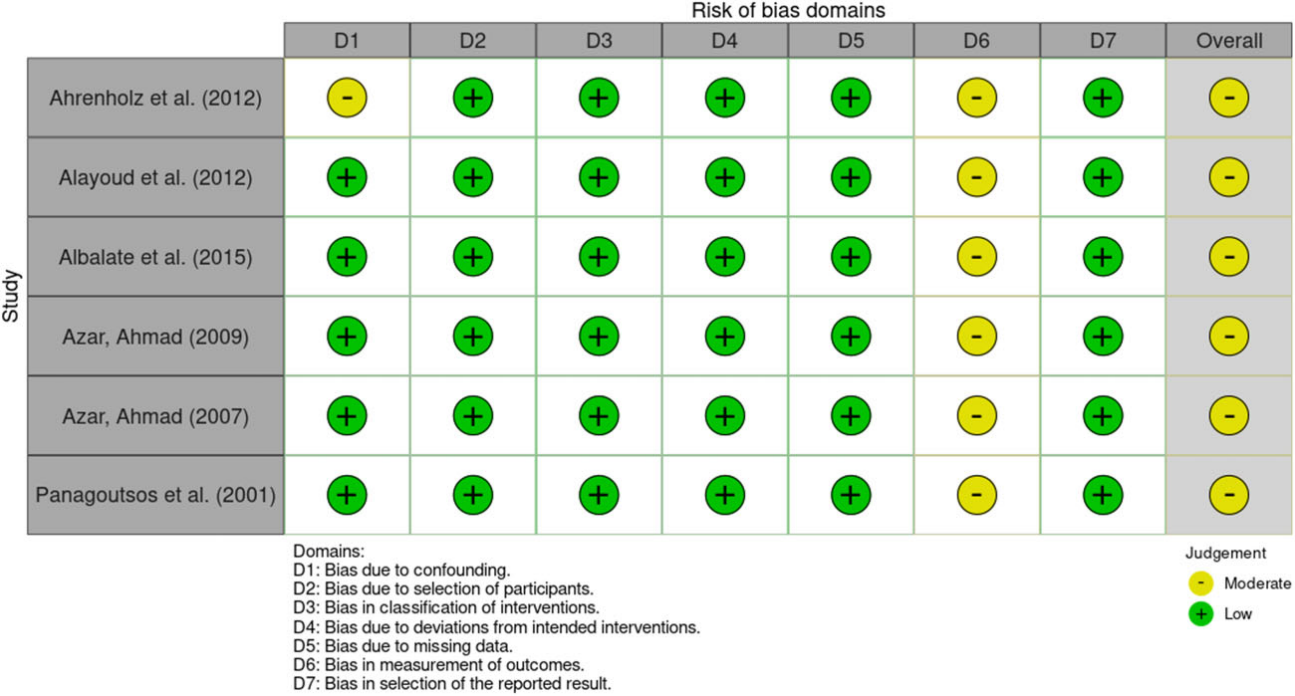
Risk of bias assessment for non-randomized trials using the ROBINS-I risk of bias tool.

Of the nine studies included in the review, only one was found to be at a low risk of bias (Figs. 2 and 3). Of the three RCTs included, none disclosed allocation concealment or whether participants and personnel were blinded. This led to one of the included studies being categorized as high risk of bias. The remaining seven were categorized as moderate risk due to a lack of or unclear information being presented in one or more categories.

### Meta-analysis

Only four studies [7, 27, 30, 33] were selected for meta-analysis, as the other five did not include dialysate flow rate comparisons of 300 versus 500 or 500 versus 800 ml/min [28, 29, 31, 32, 34].

### High versus standard flow rate and URR

**Figure 4:**
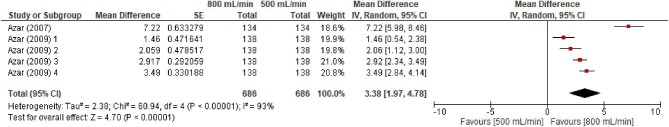
Random effects meta-analysis for URR—500 ml/min versus 800 ml/min.

Two studies with a total of five comparisons were included in the meta-analysis comparing URR for dialysate flow rates of 500 and 800 ml/min [7, 30] (Fig. 4). A dialysate flow rate of 800 ml/min increased URR by 3.38 (95% CI 1.97–4.78, *P* < .00001). Heterogeneity was present as per the *I*^2^ statistic (93%) and the χ^2^ test (*P* < .00001).

### High versus standard flow rate and spKt/V

**Figure 5:**
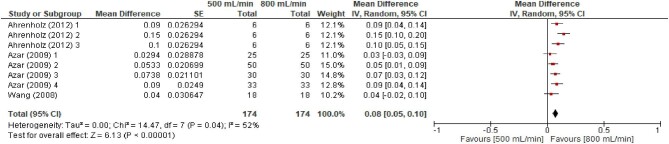
Random effects meta-analysis for spKt/V—500 ml/min versus 800 ml/min.

Three studies with a total of eight comparisons were included in our meta-analysis comparing spKt/V for dialysate flow rates of 500 ml/min and 800 ml/min [7, 27, 33] (Fig. 5). The mean difference was 0.08 (95% CI 0.05–0.10, *P* < .00001) in favour of a dialysate flow rate of 800 ml/min. The associated *I*^2^ statistic was 52% with a χ^2^ test *P*-value <.04, suggesting a moderate degree of heterogeneity.

### Sensitivity analysis

Sensitivity analysis results considering correlation coefficients of 0.25 are presented in Supplementary Figs. 1 and 2. Comparing 500 ml/min versus 800 ml/min, assuming a correlation coefficient of 0.25, we found mean differences of 3.33 URR (95% CI 1.93–4.73) and 0.08 spKt/V (95% CI 0.05–0.10), both statistically significant in favour of 800 ml/min. In Supplementary Figs. 3 and 4, sensitivity analyses results assuming a correlation coefficient of 0.9 found mean differences of 3.41 URR (95% CI 1.98–4.85) and 0.08 spKt/V (95% CI 0.05–0.10), both statistically significant in favour of 800 ml/min. These results are both similar in direction and magnitude to those found in the primary analyses.

### Included studies

Four studies that were excluded from the meta-analysis [28, 29, 31, 32] reported that an increase in dialysate flow rate was associated with an increase in Kt/V (unspecified type) and/or URR. Alayoud *et al*. [28] compared 500 ml/min versus 700 ml/min in 33 patients and found that the means and SDs for Kt/V (as measured via online clearance monitoring) modestly increased from 1.50 ± 0.16 to 1.52 ± 0.16. Similarly, Molano-Trivano *et al*. [31] found that Kt/V increased from 1.57 ± 0.25 to 1.59 ± 0.23 when comparing 400 ml/min versus 500 ml/min for a population of 100 patients. Albalate *et al*. [29] showed that in 31 patients there was a 4% increase in Kt when comparing 400 ml/min versus 500 ml/min and an additional 3% increase in Kt when comparing 500 ml/min versus 700 ml/min. Panagoutsos *et al*. [32] compared 500 ml/min versus 560 ml/min in 34 patients and found that URR increased from 52.8 ± 8 to 71 ± 7 and Kt/V increased from 0.93 ± 0.19 to 1.55 ± 0.29. It is important to note that throughout the duration of this 2-year trial, blood flow increased from 240 ml/min to 410 ml/min, which may have had a significant impact on these results.

### Secondary outcomes

Only one of the studies with 18 participants included a secondary outcome of interest [33]. It compared results from the KDQoL-SF questionnaire as well as the Health Utility Index Mark 2 (HUI2) questionnaire for health-related quality of life (HRQoL) in patients on HD whose dialysate flow was 500 ml/min versus 800 ml/min. The results showed that there was no difference in KDQoL [mean difference −5.25 (95% CI −15.00–4.50)] or HUI2 [mean difference −0.04 (95% CI −0.16–0.08)] between the two groups. No other study included HRQoL, symptom burden, cognition, physical function or mortality.

### Exploratory analysis

Four studies with a total of 15 comparisons were included in the network meta-analysis [7, 27, 33, 34] comparing spKt/V for dialysate flow rates of 300, 600 and 800 ml/min with 500 ml/min (four treatments). The 300, 600 and 800 ml/min dialysate flow rates were associated with statistically significant mean differences of −0.18 spKt/V (95% CI −0.22 to −0.15), 0.11 spKt/V (95% CI 0.02–0.19) and 0.08 spKt/V (95% CI 0.06–0.10), respectively, compared with the standard dialysate flow rate (500 ml/min). Although moderate heterogeneity was present, the associated CI was wide [*I*^2^ = 40.6% (95% CI 0.0–71.6), *P* = .0871). As such, inferences regarding the extent of heterogeneity should be made with caution. A visual representation of the network is presented in Supplementary Fig. 5, with results presented in Supplementary Fig. 6 and Supplementary Table 1.

## DISCUSSION

In this systematic review of nine studies and meta-analysis of four studies involving 576 adult patients with kidney failure receiving maintenance HD, we found that increasing dialysate flow rates from 500 ml/min to 800 ml/min improved dialysis adequacy as measured by URR (3.38) and spKt/V (0.08). We also found in an exploratory analysis that increasing dialysate flow rates from 300 ml/min to 500 ml/min improved spKt/V by 0.18. There were limited data on other patient-important outcomes, so the impact of increasing dialysis flow rates on symptoms, cognition, physical function and quality of life is unclear. Results of the exploratory analysis generally supported those found in the primary analyses with respect to the positive relationship between dialysate flow rate and dialysis adequacy as measured by spKt/V.

This study addresses an important question with regards to the effectiveness of increasing dialysis flow rates to improve dialysis adequacy and its impact on patient-important outcomes in the clinical HD setting. Although dialyzer specification sheets provide information about small molecule clearance (e.g. urea, phosphate, creatinine, vitamin B12) across a range of dialysate flow rates *in vitro*, this systematic review and meta-analysis is the first of its kind to summarize the literature *in vivo* across a range a settings, populations and HD prescriptions. For example, an *in vitro* study of dialyzer mass transfer area coefficient (K_o_A) for urea was examined in 22 different models of commercial hollow fibre dialyzers (≈5 per model, total = 107) showed that K_o_A did not differ by blood flow, but when dialysate flow increased from 504 (6) to 819 (8) ml/min, urea K_o_A increased by 14 (7%) (range 3–33%) depending on the dialyzer model [35]. Kt/V targets are important to establish, as inadequate dialysis is associated with morbidity and mortality [5, 6, 36, 37]. A study in the USA found that many patients receiving facility-based HD were not meeting Kt/V targets. Underprescription of dialysis was identified as one of the barriers in this study, as many nephrologists were unaware of underdialysis because they did not explicitly calculate prescribed Kt/V [38]. Common aetiologies leading to underdialysis included vascular access dysfunction and inadequate HD treatment duration [39]. The updated 2015 Kidney Disease Outcomes Quality Initiative clinical practice guideline recommends a spKt/V target of 1.4 per HD session for patients treated thrice weekly, with a minimum delivered spKt/V of 1.2 (1b recommendation) that may be reduced in patients with significant residual native kidney function (not graded) [40]. A stdKt/V target of 2.3 per week with a minimum delivered stdKt/V of 2.1 is suggested in patients on HD schedules other than thrice weekly with incorporation of ultrafiltration or residual kidney function (not graded).

Kt/V targets may be achieved by altering many different aspects of the HD prescription, including dialysis frequency, duration, blood flow rate, dialysate flow rate, dialyzer size (including the use of dual dialyzers in parallel or series) and intradialytic exercise, but not dialysate temperature [41–44]. The relative contribution of each of these individual components and how they should be prioritized sequentially is unclear [7], but the HD prescription should be individualized for every patient depending on his/her needs, values and preferences, in addition to other system factors including facility scheduling availability, permitted duration of HD sessions, dialyzer costs and availability of intradialytic exercise [45]. A functional fistula, graft or central venous catheter is critical to optimize blood flow rates without any significant recirculation. Quality improvement programs consisting of feedback, educational workshops and materials and monitoring of dialysis time, dialyzer type, blood flow, dialysate flow and technical assistance have been shown to improve documented review of the dialysis prescription, dialysis at the prescribed treatment time and URR [46].

However, there are many barriers to increasing dialysis treatment time, including transportation; social issues; intradialytic symptoms such as cramping, nausea/vomiting and headache; personal preference and non-adherence [47, 48]. Using dialyzers with a larger surface area is common, especially in men, obesity and other settings with larger volume density, but this may still not be adequate in select patients depending on their HD prescription. Increasing dialysis flow rates from the common standard of 500 ml/min to 800 ml/min could increase spKt/V by 0.08 (95% CI 0.06–0.11) and URR by 3.38 (95% CI 1.97–4.78), with heterogeneity presumably explained on the basis of centre- and patient-related HD prescription factors [49, 50]. However, this benefit must be balanced by the economic and environmental impacts of increased dialysate flow rates. Assuming an HD session time of 4 hours, increasing the dialysate flow rate from 500 ml/min to 800 ml/min would result in an additional use of 72 l of dialysate per treatment and would result in >10 000 l of additional dialysate per year assuming a thrice weekly HD schedule [19]. Given the limited access to water in some countries that is anticipated to be exacerbated globally as climate change progresses, this may not be sustainable on a renal program level or even individual patient level from an environmental perspective unless reverse osmosis water is recycled or reused and other green nephrology initiatives are adopted [51–53]. Lastly, depending on the volume of dialysate required per HD session, consumable costs may be significantly increased as a result of additional bicarbonate or acid concentrates for online generation of dialysate, adding dialysis waste [54, 55].

It should be noted that there is a lack of literature comparing dialysis adequacy associated with a 600 ml/min dialysate flow rate versus 300, 500 or 800 ml/min. As such, there were limited direct comparisons for 600 ml/min in our exploratory network meta-analysis, contributing to low confidence in the point estimates. Nonetheless, the conclusions drawn from the exploratory analysis generally support those of the main analyses, suggesting a positive relationship between dialysate flow rate and adequacy.

The strengths of this systematic review and meta-analysis include its novelty, comprehensive search strategy developed by a medical librarian, inclusion of only RCTs where only dialysate flow rates were modified and other elements of the HD prescription were fixed, limiting confounding, and its focus on widely used clinical benchmarks of low, standard and high dialysate flow rates. Limitations include a small number of studies with small sample sizes and limited reporting of patient-important outcome measures related to dialysis adequacy such as HRQOL and symptom burden and no reporting of other markers of dialysis adequacy such as clearance of β2 microglobulin and other small or middle molecules. In addition, studies were low quality at a high or moderate risk of bias due to underreporting of randomization sequence generation, allocation concealment and blinding methods. Because there were no studies in a home HD population, the generalizability of our findings may not extend beyond facility-based HD. Furthermore, because of the small number of studies, we were unable to perform a meta-regression across the spectrum of HD prescriptions and therefore we cannot comment on the generalizability of the results to specific types of HD prescriptions. Finally, heterogeneity in results is likely explained by different study settings populations, HD prescriptions and urea kinetic modelling approaches that we were unable to explore in meta-regression due to the limited number of studies [56–60].

In summary, increasing dialysis flow rates in adults receiving maintenance HD improves dialysis adequacy as measured by urea kinetic modelling metrics including URR and spKt/V. The impact of these improvements on patient-important outcomes and the economic and environmental impact of increasing dialysis flow rates to 800 ml/min as compared with other strategies to increase dialysis adequacy needs further evaluation in high-quality prospective studies.

## Supplementary Material

sfae163_Supplemental_File

## Data Availability

The data underlying this article are available in the article itself.
