# Supplementary material for: The impact of dialysate flow rate on haemodialysis adequacy: a systematic review and meta-analysis
Source: Clin Kidney J. 2024 Jun 4;17(7):sfae163. doi: 10.1093/ckj/sfae163 (PMC11229034; doi:10.1093/ckj/sfae163)
Supplement: sfae163_Supplemental_File [file sfae163_supplemental_file.docx]

**Supplemental:**

Search strategy: In collaboration with a medical librarian, original research articles were identified from the following databases: Medline, Embase, and CENTRAL (Cochrane Library). The search strategy was tailored to each database and used a combination of key terms. The grey literature was not systematically assessed. Included studies were randomized controlled trials and observational trials without any limits on sample size.

**Supplemental Item 1: Medline search strategy and results**

('dialyzer clearance'[Title] AND ('flow rate'[Title] OR 'flow rates'[Title])) OR (('dialysis'[Title] OR 'haemodialysis'[Title] OR  'hemodialysis'[Title]) AND (adequacy[Title] OR 'clearance'[Title])) OR 'urea reduction ratio' OR ('kt/v'[Title] AND (dialysis[Title] or dialyzer[Title] OR dialysate[Title] OR hemodialysis[Title] OR haemodialysis[Title])) OR 'haemodialysis dose'[Title] OR 'dialysis dose'[Title] OR 'dialysate flow'[Title] Filters: from 1995 - 2022  *2358 hits*

**Supplemental Item 2: Central (the Cochrane Library) search strategy and results**

('dialyzer clearanc*':ti AND 'flow rate*':ti) OR (('dialysis' OR 'hemodialysis') NEAR (adequacy OR 'clearance')):ti OR 'urea reduction ratio':ti,ab OR ('kt*v' AND (dialys* OR hemodialys*)):ti,ab OR 'haemodialysis dose':ti OR 'dialysis dose':ti OR 'dialysate flow':ti  *453 hits*

**Supplemental Figure 1: Sensitivity analysis: random effects meta-analysis for spKt/V – 500 mL/min vs 800 mL/min, correlation coefficient 0.25**


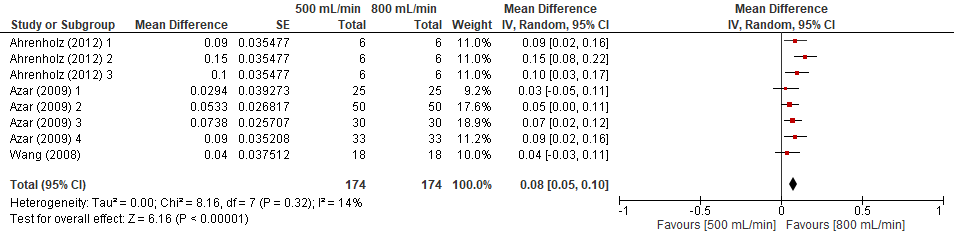


**Supplemental Figure 2: Sensitivity analysis: random effects meta-analysis for URR – 500 mL/min vs 800 mL/min, correlation coefficient 0.25**


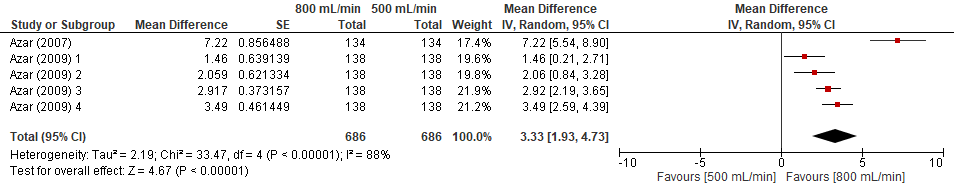


**Supplemental Figure 3: Sensitivity analysis: random effects meta-analysis for spKt/V – 500 mL/min vs 800 mL/min, correlation coefficient 0.9**


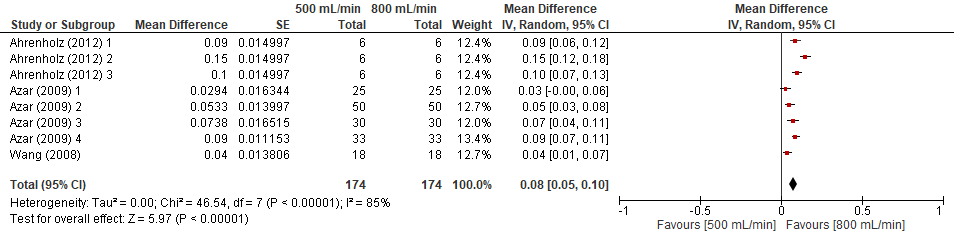


**Supplemental Figure 4: Sensitivity analysis: random effects meta-analysis for URR – 500 mL/min vs 800 mL/min, correlation coefficient 0.9**


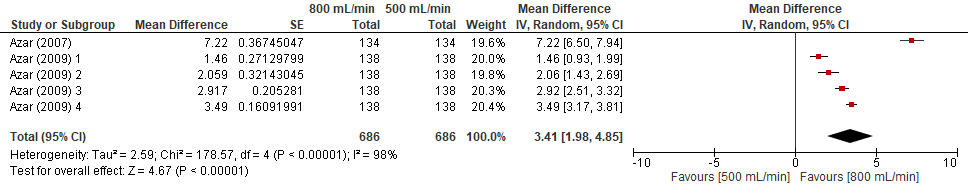


**Supplemental Table 1: Random effects network meta-analysis for spKt/V – 500 mL/min vs 300, 60 and 800 mL/min**

| **Treatment** | **Mean Difference** | **95% CI** | **Z** | **P-value** |
| --- | --- | --- | --- | --- |
| 300 mL/min | -0.18 | -0.22 to -0.15 | -10.15 | <0.0001 |
| 500 mL/min (reference) | - | - | - | - |
| 600 mL/min | 0.11 | 0.02 to 0.19 | 2.52 | 0.0117 |
| 800 mL/min | 0.08 | 0.06 to 0.10 | 6.78 | <0.0001 |

**Supplemental Figure 5: Network Graph - random effects network meta-analysis for spKt/V – 500 mL/min vs 300, 600 and 800 mL/min**

**
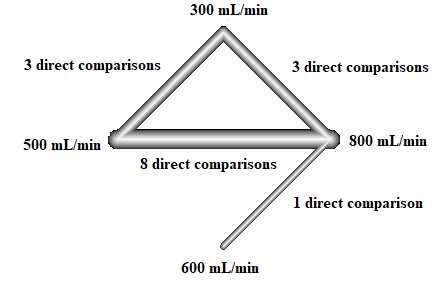
**

**Supplemental Figure 6: Random effects network meta-analysis for spKt/V – 500 mL/min vs 300, 600 and 800 mL/min**

**
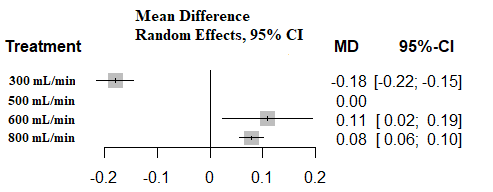
**
